# Clinical efficacy of simethicone combined with bifidobacterium in the treatment of pediatric aerophagia

**DOI:** 10.3389/fped.2025.1607826

**Published:** 2025-08-14

**Authors:** Ping He, Fangqi Hu, Le Wang

**Affiliations:** Department of Pediatrics, Anqing Municipal Hospital, Anqing, Anhui, China

**Keywords:** simethicone, bifidobacterium, aerophagia, children, treat

## Abstract

**Background:**

The causes of children aerophagia are complex, and the treatment methods are diverse. Moreover, it is prone to recurrent attacks. Simethicone oil, as a safe and new type of drug, can be used for treatment.To evaluate the clinical efficacy of simethicone combined with bifidobacterium for the treatment of pediatric aerophagia.

**Methods:**

This study evaluated two treatment approaches for aerophagia. The control group received monotherapy with Bifidobacterium, while the treatment group received combination therapy with Bifidobacterium and simethicone. Clinical efficacy, gastrointestinal symptom scores (GSRS), gastric motility parameters, and adverse events were compared between the two groups.

**Results:**

Among the 80 children with aerophagia, 14 (17.5%) were preschool children, 56 (70%) were school-aged children and 10 (12.5%) were adolescents children. Seventy children (87.5%) had abdominal distension as their chief complaint. The treatment group exhibited a significantly higher clinical efficacy rate (92.5% vs. 75%, *P* < 0.05) and lower post-treatment GSRS scores (*P* < 0.05). Gastric emptying rates at 60 and 120 min were also significantly improved in the treatment group (*P* < 0.05), although no significant differences were observed in the serum gastrin or plasma motilin levels (*P* > 0.05).The rate of adverse events was 7.5% in the treatment group and 15% in the control group, with no statistically significant difference (*P* > 0.05).

**Conclusion:**

The combination of simethicone and bifidobacterium enhances gastric emptying, alleviates the clinical symptoms of pediatric aerophagia, and demonstrates favorable safety and tolerability, thereby supporting its clinical application.

## 1 Introduction

Aerophagia, characterized by repetitive air swallowing leading to gastrointestinal symptoms such as bloating, belching, excessive flatulence, and abdominal pain, affects approximately 3.66% of children, with a higher prevalence in Asian populations (1). It is classified as a functional gastrointestinal disorder under the Rome IV criteria (2), but its etiology remains unclear. The current management strategies focus on symptomatic relief, psychological interventions, and pharmacological approaches. Emerging multidisciplinary strategies emphasize collaboration with pediatricians, family education, and behavioral modifications (3). Bifidobacterium, a key probiotic, modulates immune and digestive functions and has demonstrated efficacy in the treatment of functional dyspepsia (4). Simethicone, a non-absorbable antifoaming agent, reduces the surface tension of gas bubbles in the gastrointestinal tract, facilitating gas expulsion and alleviating bloating. It is widely utilized in pediatric endoscopy (5) and can be regarded as a type of drug similar to prokinetic agents to enhance gastric emptying (6, 7). However, the chronic and recurrent nature of aerophagia often limits the efficacy of monotherapies. This study investigated the synergistic effects of simethicone combined with bifidobacterium in pediatric aerophagia with the aim of establishing a more effective therapeutic regimen.

## 2 Methods

### 2.1 Participants

Children meeting Rome IV criteria for aerophagia (2) were screened for eligibility. Potential participants underwent screening based on predefined inclusion and exclusion criteria. The inclusion criteria were as follows: (1) recurrent air swallowing with significant gastrointestinal gas accumulation; (2) worsening daytime bloating with meals; (3) frequent belching/flatulence. Symptoms must persist for ≥6 months, with active criteria met in the preceding 3 months. The exclusion criteria included organic gastrointestinal or systemic diseases. Participants meeting all inclusion criteria and none of the exclusion criteria were eligible for enrollment and subsequent randomization into the study groups. This study was approved by the Ethics Committee of our hospital.

### 2.2 Treatment protocols

Control Group: Oral bifidobacterium (105–420 mg/dose, twice daily, this product is a live bacterial preparation composed of long-type bifidobacteria, lactobacillus acidophilus, enterococcus faecalis, and is appropriately combined to form a main component being bifidobacteria. Made in Shanghai Xinyi Pharmaceutical Factory Co., Ltd) post-meals.Treatment Group: Bifidobacterium + simethicone emulsion (1–2 ml/dose, thrice daily. Made in Zhejiang Saimo Pharmaceutical Co., LTD).Both groups received 1-week of treatment.For 3 days prior to the treatment, drugs that alter the acid-base balance in the stomach or affect gastrointestinal motility should be avoided.

### 2.3 Monitoring indicators

#### 2.3.1 Clinical efficacy assessment

Therapeutic outcomes were evaluated using a three-tier efficacy classification: Markedly effective: Significant symptom resolution requiring no further intervention. Effective: Symptom improvement with no functional impairment to daily activities or studies. Ineffective: No symptom improvement or persistent need for additional treatment.

#### 2.3.2 GSRS scores

Gastrointestinal symptoms were quantified using the Gastrointestinal Symptom Rating Scale (GSRS) (8).

#### 2.3.3 Gastric motility assessment

Gastric emptying rate: Measured by ultrasound after 1-week treatment. Protocol: After 3–4 h fasting, patients will consume 300 ml milk within 5 minutes. Antrum cross-sectional area will be scanned at 60/120 min postprandially. Gastrointestinal hormones: Serum gastrin and plasma motilin levels will be assayed pre-treatment and post-treatment.

#### 2.3.4 Adverse events monitoring

The following events were systematically recorded: Gastrointestinal discomfort (abdominal pain, nausea, diarrhea, constipation, anorexia); rash; dizziness.

### 2.4 Statistical analysis

Data were analyzed using SPSS 20.0. Continuous variables (mean ± SD) were compared using t-tests and categorical variables (%) were compared using chi-square tests. Statistical significance was set at *P* < 0.05.

## 3 Results

### 3.1 Baseline characteristics

Between February 2023 and February 2025, ninety-two children with aerophagia were initially enrolled. After screening, twelve were excluded for the following reasons: six failed to meet inclusion criteria, five declined participation, and one withdrew against medical advice. A total of eighty children were randomized into two groups. Among the eighty children with aerophagia, there were twenty-eight males and twelve females in the control group, aged 1.5–13.2 years, with an average age of 7.45 ± 1.12 years. The treatment group included twenty-five males and fifteen females, aged 1.7–12.8 years, with an average age of (6.98 ± 0.92) years. Among the 80 children, 14 (17.5%) were preschool children, 56 (70%) were school-aged children and 10 (12.5%) were adolescents children. Seventy children (87.5%) had abdominal distension as their chief complaint, and the main clinical symptoms were increased anal exhaust, belching and air swallowing. Other gastrointestinal symptoms include retching, anorexia, abdominal pain and constipation. Among them, eight cases were combined with tic disorders. Children with aerophagia showed obvious flatulence in the intestinal cavity on abdominal ultrasound and abdominal plain film. Gastroscopy, electroencephalography and other examinations were performed in some children, and no abnormalities were found.

### 3.2 Clinical efficacy

The total efficacy rate was 75% in the control group and 92.5% in treatment group. Compared to the control group, the total effective rate of simethicone oil was higher, and the comparison between the two groups was statistically significant (*P* < 0.05) (Table 1).

**Table 1:** Analysis of total efficacy in two groups [*n* (%)].

Group	Marked effect	Effective	No effect	Total efficacy (%)
Control (*n* = 40)	9 (22.5)	21 (52.5)	10 (25.0)	30 (75.0)
Treatment (*n* = 40)	11 (27.5)	26 (65.0)	3 (7.5)	37 (92.5)
X^2^ value				4.50
*P*-value				0.034^a^

*P* < 0.05 represents statistically significance.

^a^ *P* < 0.05 vs. control group.

### 3.3 Symptom improvement

Compared with before treatment, the clinical symptoms of the two groups were improved after treatment (*P* < 0.05). At the same time, the GSRS score after simethicone treatment was lower, and the difference between the two groups was statistically significant (*P* < 0.05) (Table 2).

**Table 2:** Improvement of clinical symptoms before and after treatment [GSRS score $\bar{x}\pm s$].

Group	Pre-treatment	Post-treatmen	t-value	*P*-value
Control (*n* = 40)	15.25 ± 5.74	4.25 ± 1.92	9.98	<0.05
Treatment (*n* = 40)	14.25 ± 4.05	2.74 ± 1.77	14.61	<0.001
t-value	−0.64	1.78		
*P*-value	0.16	0.035^a^		

*P* < 0.05 represents statistically significance.

^a^ *P* < 0.05 vs. control group.

### 3.4 Gastric motility

We found that the gastric emptying rate (60 min, 120 min) of simethicone was higher than that of the control group (*P* < 0.05). However, there was no significant difference in the serum gastrin and plasma motilin levels between the two groups (*P* > 0.05) (Table 3).

**Table 3:** Evaluation of gastric motility function in two groups ($\bar{x}\pm s$).

Group	Gastric emptying rate (60 min)	Gastric emptying rate (120 min)	Serum gastrin (pg/ml)	Plasma motilin (pg/ml)
Control (*n* = 40)	54.76 ± 3.94	79.65 ± 4.40	90.2 ± 14.6	401.8 ± 50.5
Treatment (*n* = 40)	57.04 ± 3.77	83.20 ± 3.72	92.6 ± 16.8	395.7 ± 48.4
t-value	2.83	2.76	3.84	4.62
*P*-value	0.021^a^	0.014^a^	0.18^b^	0.22^b^

*P* < 0.05 represents statistically significance.

^a^ *P* < 0.05 vs. control group.

^b^ *P* > 0.05 vs. control group.

### 3.5 Safety

The incidence of adverse reactions was 7.5% in the treatment group and 15% in the control group. The total incidence of adverse reactions in the treatment group was slightly higher than that in the control group, but the difference between the two groups was not statistically significant (*P* > 0.05) (Table 4).

**Table 4:** Comparison of adverse reactions between two groups [*n*(%)].

Group	Gastrointestinal discomfort	Rash	Dizziness	Total incidence
Control (*n* = 40)	3 (7.50)	0 (0.0)	0 (0.0)	3 (7.50)
Treatment (*n* = 40)	4 (10.00)	1 (2.50)	1 (2.50)	6 (15.0)
X^2^ value				1.77
*P*-value				0.18^a^

*P* < 0.05 represents statistically significance.

^a^ *P* > 0.05 vs. control group.

## 4 Discussion

In recent years, the incidence of aerophagia (excessive air swallowing) among Chinese children has shown a progressive upward trend, attributable to escalating academic pressure and irregular dietary patterns (1). The symptomatology of this condition imposes substantial psychological burdens on affected children and their families while significantly impairing school-related quality of life. Chronic manifestations may potentially have detrimental effects on long-term developmental outcomes (9).

The current etiological understanding remains inconclusive, although aerophagia is generally classified as a functional or behavioral disorder that may occur in otherwise healthy children or those with underlying medical conditions (3, 10). The clinical presentation is heterogeneous with predominant symptoms including abdominal distension, excessive flatulence, belching, and abdominal pain secondary to excessive air ingestion. Physical examination typically reveals tympanic and hyperactive abdominal bowel sounds. In our case series, 70 (87.5%) chlidren presented with abdominal distension. This symptom demonstrates diurnal variation characterized by a “morning mildness and evening exacerbation” pattern, correlating with cumulative air swallowing and postprandial aggravation, even some of the patients presented to the hospital merely with severe abdominal distension as their symptom (11), ultimately resulting in significantly diminished quality of life compared to healthy peers (12). The rising adolescent incidence parallels societal development trends and frequent comorbidities with tic disorders, suggesting potential neuropsychiatric associations with conditions such as Tourette syndrome (TS) (13). Our observations the corroborate concurrent tic manifestations including ocular blinking, throat clearing, and cervical tics in some children. Furthermore, Oswari et al. identified significant correlations between familial stress and aerophagia (14) with psychological trauma, emotional stressors, and atypical parenting practices that potentially trigger or exacerbate symptoms. Notably, aerophagia may occasionally present as a primary manifestation of depressive disorders in pediatric populations (15).

The diagnosis of aerophagia is mainly based on symptom manifestations, medical history collection, and the exclusion of other organic diseases (2). The children with aerophagia selected for this study also underwent relevant tests, such as abdominal upright radiographs, gastroscopy, and gastrointestinal contrast imaging. There are various treatment methods for aerophagia, including psychological counseling, dietary adjustment and medication (2, 16, 17). However, persistent recurrence and severe complications,such as protracted nausea and abdominal distension,remain clinical challenges despite intervention (18). While bifidobacterium,an essential intestinal probiotic,has been widely applied in the management of functional dyspepsia (19), current evidence suggests negligible differential efficacy compared with placebo in pain alleviation. Simethicone, a routine antifoaming agent used in pediatric endoscopy (20), demonstrates antidistension properties and gastrointestinal mucosal protection through bubble reduction, facilitation of gastric emptying, and mitigation of postprandial fullness. Our study revealed that simethicone monotherapy exhibited superior efficacy compared with controls after 1 week, with significantly greater symptomatic improvement in the treatment group. Notably, combination therapy with simethicone and bifidobacterium enhanced gastric emptying rates without altering gastrin or motilin levels compared with controls, indicating prokinetic effects independent of hormonal modulation and favorable safety profiles. Adverse event analysis indicated comparable rates of mild adverse reactions between the combination and monotherapy groups, consistent with the existing safety data for simethicone in dyspepsia management (21).

In conclusion, simethicone-bifidobacterium combination therapy effectively enhanced gastric motility, alleviated clinical symptoms, and demonstrated satisfactory safety and tolerability in pediatric aerophagia, warranting a broader clinical application.

## Data Availability

The original contributions presented in the study are included in the article/Supplementary Material, further inquiries can be directed to the corresponding author.
